# Associations between lifestyle intervention‐related changes in dietary targets and migraine headaches among women in the Women's Health and Migraine (WHAM) randomized controlled trial

**DOI:** 10.1002/osp4.376

**Published:** 2020-02-08

**Authors:** Whitney E. Evans, Hollie A. Raynor, Whitney Howie, Richard B. Lipton, Graham J. Thomas, Rena R. Wing, Jelena Pavlovic, Samantha G. Farris, Dale S. Bond

**Affiliations:** ^1^ Weight Control and Diabetes Research Center Brown University Alpert Medical School/The Miriam Hospital Providence Rhode Island USA; ^2^ Department of Nutrition University of Tennessee Knoxville Tennessee; ^3^ Department of Neurology Albert Einstein College of Medicine/Montefiore Medical Center New York USA; ^4^ Department of Psychology, Rutgers the State University of New Jersey Piscataway New Jersey

**Keywords:** diet, migraine, obesity, weight loss

## Abstract

**Background:**

Migraine and obesity are comorbid particularly in women of reproductive age. Obesity treatment involves reducing energy intake and improving dietary quality but the effect of these changes on migraine is largely unknown.

**Objective:**

To determine if adherence to dietary intervention targets (ie, total energy, dietary fat intake, and dietary quality) were associated with improvements in migraine and weight.

**Methods:**

Eighty‐four women with overweight/obesity and migraine were randomized to and completed either a 16‐week behavioral weight loss (BWL) or a migraine education (ME) intervention. For 28 days at baseline and posttreatment, women recorded monthly migraine days, duration, and maximum pain intensity via smartphone‐based diary. At each assessment, weight was measured and dietary intake (total energy intake, percent (%) energy from fat, and diet quality, as measured by the Healthy Eating Index, 2010 [HEI‐2010]) was assessed using three nonconsecutive 24‐hour diet recalls.

**Results:**

There were no significant group differences in change mean migraine days per month (BWL: ‐2.6+4.0, ME: ‐4.0+4.4; *p* = 0.1). Participants in BWL significantly reduced their percent fat intake 3.8% (*p* = 0.004) and improved total diet quality (HEI‐2010) by 6.7 points (*p* = 0.003) relative to baseline and those in ME (%fat: +0.3%; *p* = 0.821; HEI‐2010: +0.7; *p* = 0.725). After controlling for race/ethnicity and weight change, changes in dietary intake were not related to changes in migraine characteristics or weight loss among BWL participants (*p*'s > 0.05).

**Conclusions:**

Changes in dietary intake among participants were small and may have been insufficient to improve migraine in women with overweight/obesity and migraine.

## 1 INTRODUCTION

Migraine is comorbid with obesity, particularly in women of reproductive age.^1^, ^2^, ^3^, ^4^ Among women with obesity, the odds of migraine is 37% to 81% higher than among women with a healthy BMI.^5^, ^6^ Mechanisms underlying this comorbidity are not fully understood; however, it is hypothesized that the chronic inflammatory state of obesity may heighten the neurovascular inflammatory response in migraine.^2^, ^3^, ^7^, ^8^ Indeed, an energy‐dense nutrient‐poor diet, which is implicated in both migraine and obesity, contributes to systemic inflammation and therefore represents a potential modifiable risk factor that could be used to simultaneously address both diseases. 7, 9, 10, 11, 12 While reducing energy intake is the primary recommendation for weight loss, specific dietary recommendations for migraine management are less certain.^13^, ^14^, ^15^

Perhaps the most popular dietary approach to migraine management is avoidance of specific dietary triggers including chocolate, cheese, processed meats, red wine, caffeine, artificial sweeteners, and sodium.^11^, ^14^, ^15^, ^16^ Nearly one‐third of adults with migraine self‐report diet‐related triggers, although there is considerable variability across individuals.^17^ A recent examination of triggers at the individual level showed that individual triggers are unique in up to 85% of patients.^18^ Thus, while following an elimination diet is associated with a reduction in migraine attacks, this type of dietary approach has low adherence and limited generalizability given the interindividual variation in triggers.^19^, ^20^

Another popular approach to migraine management is following a specific diet, such as the ketogenic or low‐fat diets. While the ketogenic diet has shown some promise in reducing migraine frequency, this high‐fat very low‐carbohydrate diet is difficult to follow and is often deficient in fiber and other key nutrients.^21^, ^22^ Similarly, while one trial found that following a low‐fat diet (<20% energy from fat) was associated with significant reductions in migraine frequency and severity when compared to a diet with normal fat content (25%‐30% energy from fat), another trial found that following a low‐fat vegan diet had no significant effect on migraine frequency, severity, and duration but reduced migraine‐related pain.^23^, ^24^ Given the limited research that has been done to examine the efficacy of specific dietary approaches for migraine management, there is no widely accepted dietary prescription.^11^ Therefore, studies that (1) examine the effect of specific dietary prescriptions on migraine management, (2) test their efficacy in the context of a randomized controlled trial, and (3) assess dietary adherence through repeat dietary assessments are needed.

The Women's Health and Migraine (WHAM) study is a randomized controlled trial that tested the impact of a standardized behavioral weight loss (BWL) intervention versus migraine education (ME) control on migraine frequency and indices of severity in women who had both migraine and overweight/obesity.^25^ Although BWL achieved a significantly greater mean ± SE weight loss than ME (−3.8±2.5 kg vs +0.9±0.9 kg; *p* < 0.001) at 16‐week posttreatment (primary endpoint), both groups experienced significant improvements in monthly mean ± SE migraine days posttreatment (−3.0±2.0 vs −4.0±4.0). Similar patterns were observed for other migraine severity indices.^25^

Given that WHAM participants assigned to BWL were prescribed a standard low‐fat low calorie diet and provided strategies to improve dietary quality as per USDA guidelines, the WHAM trial provides a unique opportunity to evaluate whether adherence to this dietary approach is associated with greater improvements in migraine and/or weight loss compared to a control intervention. Findings have the potential to provide greater insight into the role of diet in the migraine and obesity link and have implications for informing development of dietary interventions to concurrently target both diseases.

This post hoc exploratory completers analysis evaluated (1) whether women who were randomized to and completed BWL had greater changes in dietary intervention targets (ie, total energy, dietary fat intake and dietary quality) than those who completed ME and (2) whether changes in dietary intervention targets were associated with migraine improvements in BWL participant completers.

## 2 METHODS

### 2.1 Participants

Participants were recruited from the Providence, RI area using direct mailings, advertisements, and social media postings between November 2012 and June 2016. Interested participants completed a telephone screen and were invited to an in‐person orientation where the study details were provided, and migraine diagnosis was confirmed by a study neurologist. Eligible participants were women, ages 18 to 50 years old, with overweight or obesity (BMI 25.0‐49.9 kg/m^2^), who had neurologist‐confirmed migraine with a frequency of three or more migraine attacks and 4 to 20 migraine headache days per month. Women with medication overuse headaches at baseline were excluded from the study, whereas those with chronic migraine (15‐20 headache days) were included. Additional details on trial inclusion and exclusion criteria and recruitment methods have been reported previously.^25^

### 2.2 Intervention

The primary objective of this parallel‐group single‐blinded randomized controlled trial (http://ClinicalTrials.gov Identifier: NCT01197196) was to compare the efficacy of two interventions (BWL or ME) on migraine frequency and severity. Participants were randomized in a 1:1 ratio using computer‐generated randomly permuted blocks of two, four, and six. Prior to randomization, participants completed informed consent and the baseline assessment, which included questionnaires, anthropometric measures, and receipt of a smartphone to record migraine headache activity for 28 consecutive days. During these four weeks, participants also completed three nonconsecutive 24‐hour diet recalls over the phone. After the initial monitoring period was complete, participants received their random assignment. Identical procedures were used to evaluate participants posttreatment (16‐20 weeks). This study was approved by The Miriam Hospital Institutional Review Board.

Participants in both conditions attended 16 weekly group meetings led by a behavioral interventionist. Participants randomized to BWL were encouraged to lose 1 to 2 pounds/week to achieve ≥7% weight loss over the intervention period. To promote weight loss, participants were prescribed a calorie‐restricted low‐fat diet with goals of 1200 to 1500 kcal/day and 33 to 42 fat grams/day (25% calories from fat) based on baseline weight, progressed to a goal of 250 minutes/week of moderate intensity exercise (50 minutes, 5 days/week), and were taught behavioral modification strategies.^25^, ^26^ Strategies taught to improve adherence to the prescribed diet included: daily recording of caloric and fat content of all food and drink consumed, limiting portion size by weighing and measuring foods, selecting prepackaged foods and eating foods prepared at home, using structured meal plans and grocery lists, and removing high‐calorie high‐fat food items from the home. BWL sessions did not include discussion of migraine or its treatment. ME was an active control condition, in that participants randomized to ME‐attended lectures based on content developed by the National Headache Foundation, which included didactic information on migraine (eg, symptoms, pathophysiology, risk factors for progression) and both standard and alternative pharmacological and nonpharmacological treatments.^27^ ME participants did not receive dietary prescriptions or behavioral strategies to produce weight loss but were provided brief education on diet and unhealthy eating as a potential risk factor for migraine progression.

### 2.3 Measures

#### 2.3.1 Migraine frequency and severity

Participants used a smartphone‐based diary application to record migraine headache occurrence (“Did you have a headache today? Yes/No”), maximum headache pain intensity (0 “no pain” to 10 “pain as bad as you can imagine”), and attack duration (hours) daily prior to bedtime for 28 consecutive days.^27^ Because data were collected via smartphone, they were transmitted to study staff for review in real time, where, if data were incomplete, research staff called the participants to inquire about the missing data. Data from the 28 days of monitoring were summarized as monthly migraine days, mean daily duration (in hours), and average maximum pain intensity.

#### 2.3.2 Demographic characteristics

At baseline, study sample characteristics were assessed via questionnaire.

#### 2.3.3 Diet

Diet was assessed via three nonconsecutive 24‐hour recalls (two weekday recalls, one weekend recall) at baseline and posttreatment. Each 24‐hour recall was collected over the phone by a trained interviewer using Nutrition Data Systems for Research (NDSR) (Version 2013, Nutrition Coordinating Center (NCC), University of Minnesota, Minneapolis, MN).^28^ NDSR employs a multiple‐pass method and a standardized portion‐size manual to decrease measurement error and bias. Through multiple passes, the details of every food or drink consumed at each identified eating occasion were reported for the previous day.

To characterize the dietary intake of women with obesity and migraine, data from the 24‐hour diet recalls collected at baseline and posttreatment were used to determine a weighted average for total energy intake, percent energy from fat, and diet quality, as measured by the Healthy Eating Index, 2010 (HEI‐2010) for both time points. Specifically, data from weekdays (Monday through Friday) versus weekend days (Saturday and Sunday) were weighted to account for 5 of 7 and 2 of 7 days of intake, respectively. While diet quality was not a specific intervention target, many of the lessons covered in BWL addressed dietary changes that would improve diet quality if followed. Accordingly, the HEI‐2010, the scoring system developed by the USDA's Center for Nutrition Policy and Promotion (CNPP) to measure degree of compliance with the 2010 Dietary Guidelines for Americans (2010 DGA), was used as a measure of total diet quality.^29^ It is composed of 12 nutrient‐ and food‐based component scores, nine of which represent adequacy components (fruit, whole fruit, total vegetables, greens and beans, whole grains, dairy, protein, seafood and plant protein, and fatty acids) and three of which represent moderation components (refined grains, sodium, and energy from solid fat, alcohol, and added sugars). Total and component HEI‐2010 scores were calculated for each 24‐hour diet recall using the unpublished *Guide to Creating Variables Needed to Calculate Scores for Each Component of the Healthy Eating Index‐2010*, (July 2013) developed by the NCC along with methodology developed for use with NDSR.^29^, ^30^ A higher HEI‐2010 total score (ranging from 0 to 100) represents greater adherence to the 2010 DGA and higher diet quality.

### 2.4 Statistical Analysis

All analyses were completed using SAS, version 9.4 (Cary, NC), at an alpha level of 0.05. Baseline demographics, anthropometrics, and migraine characteristics were summarized using the mean, standard deviation, (SD) or number with percentage. All analyses included participants who provided the primary outcomes data at both the baseline and posttreatment assessments and were repeated with only those who did not underreport energy. Specifically, given the prevalence of underreporting of energy intake in self‐reported measures of diet, underreporting of energy intake was assessed at baseline and posttreatment using the ratio of BMR:EI.^31^, ^32^, ^33^ Specifically, each participant's basal metabolic rate (BMR) was calculated using the WHO equation at each time point and compared to their reported energy intake (EI).^34^ When the BMR:EI ratio was <0.92, the recall was categorized as underreported. A sensitivity analysis was performed to compare each of the models with the full sample to only those with “reliable” recalls. A similar approach was used to examine the effect of missing dietary data. Baseline observation carried forward was used for all participants missing dietary data at follow‐up. In both instances, the pattern of results was not different, such that results from the whole sample are reported. Using a restricted maximum likelihood approach, linear mixed effects models were used to conduct between‐groups comparisons of diet (total energy intake, percent energy from fat, and total diet quality). Time, representing baseline and posttreatment, and treatment condition, BWL vs ME, were included in the models to test the significance of a group by time interaction term. Race/ethnicity (non‐Hispanic White versus all others) was controlled due to an imbalance between groups. Weight and weight change were also controlled due to the potential influence on the outcomes. Similar models were used to examine how changes in diet (total energy intake, percent energy from fat, and total diet quality entered as simultaneous predictors) related to changes in monthly migraine days, mean daily duration (hours), and average maximum pain severity in BWL participants only.

## 3 RESULTS

### 3.1 Participant characteristics

One hundred ten women were randomized to BWL (n = 54) or ME (n = 56) at baseline. At posttreatment (4 months), overall retention was 78% (n = 85). The consort diagram can be found elsewhere.^25^ In this completer's analysis, three additional participants were excluded given that they did not provide three reliable nonconsecutive recalls on two weekdays and one weekend at baseline, such that the final sample analyzed was 82: BWL (n = 41) and ME (n = 41). Of these, sufficient recalls were completed by n = 35 in BWL (85.4%) and n = 38 in ME (92.7%), for a total of n = 73 (89.0%) contributing data at posttreatment.

Demographic characteristics of BWL and ME completers are shown in **Table** 1. At baseline, participants were mean±SD 38.8 ± 8.0 years of age and had a BMI of 35.4 ± 8.2 kg/m^2^, on average. There were no statistically significant differences in migraine characteristics between BWL and ME at baseline. On average, women in both groups had mean±SD 8.2 ± 4.5 migraine days per month with an average attack lasting 18.7 ± 13.2 hours. In this subsample, there were no significant group differences in change mean migraine days per month (BWL: −2.6+4.0, ME: −4.0+4.4; *p* = 0.1).

**Table 1:** Baseline demographic characteristics and anthropometrics of women participants enrolled in the BWL and ME interventions

	Total (n = 82)	BWL^1^ (n = 41)	ME^1^ (n = 41)
**Demographic characteristics**			
Age, mean (SD), yrs	38.8 (8.0)	38.2 (7.2)	39.5 (8.6)
Race, n (%)			
African American	10 (12.2)	8	2
White	62 (75.6)	28	34
Other	10 (6.1)	5	5
Ethnicity, n (%)			
Hispanic	16 (19.5)	1 (2.4)	15 (36.6)
Not Hispanic	66 (80.5)	40 (97.6)	26 (63.4)
Education, n (%)			
High School Degree	7 (8.5)	4 (9.8)	3 (7.3)
Vocational Training	1 (1.2)	1 (2.4)	0 (0.0)
Some College	25 (30.5)	11 (26.8)	14 (34.1)
College/University Degree	33 (40.3)	18 (43.9)	15 (36.6)
Graduate/Professional Degree	16 (19.5)	7 (17.1)	9 (22.0)
Marital status, n (%)			
Married	45 (54.9)	20 (48.7)	25 (61.0)
Not married (cohabitating)	6 (7.3)	4 (9.8)	2 (4.9)
Never married	20 (24.4)	13 (31.7)	7 (17.0)
Separated or divorced	10 (12.2)	4 (9.8)	6 (14.6)
Other	1 (1.2)	0 (0.0)	1 (2.5)
BMI, mean (SD), kg/m^2^	35.4 (8.2)	36.4 (6.9)	34.3 (9.3)
Migraine Days, mean (SD)	8.2 (4.5)	7.4 (4.0)	8.9 (5.0)
Migraine Duration, mean (SD), hours	18.7 (13.2)	17.4 (9.3)	19.9 (16.3)
Max Pain Severity, mean (SD)	5.6 (1.5)	5.5 (1.5)	5.7 (1.5)
^1^BWL, behavioral weight loss; ME, migraine education			

### 3.2 Dietary intervention target assessment

As shown in **Table** 2, there was no statistically significant group by time interaction with respect to changes in reported total energy intake; participants reduced their reported total energy intake by approximately 283 calories, across groups (*p* < .001). In contrast, there were statistically significant differences in the change in percent energy from fat between the two groups from baseline to posttreatment. Specifically, BWL reduced fat intake by 3.8% (*p* = 0.004), while the 0.3% increase in ME was not significant (*p* = 0.821). Similarly, there were statistically significant differences in change in diet quality (total HEI‐2010 scores) between groups, with BWL, on average, significantly increasing their total diet quality by 6.7 units (*p* = 0.003) and ME increasing by a nonsignificant 0.7 units (*p* = 0.725).

**Table 2:** Change in reported mean ± SE dietary intake in women with migraine and obesity who completed behavioral weight loss (BWL) and migraine education (ME) interventions in the Women's Health and Migraine trial

	BWL^1^ (n = 42)		ME^1^ (n = 42)		Group* Time interaction (*p*‐value)
	*Baseline*	*Post‐Tx*	*Baseline*	*Post‐Tx*	
Energy intake (kcals/day)	1739.2 + 73.9	1402.4 + 79.4	1737.0 + 73.8	1504.3+ 76.6	*p* = 0.394
% Energy from Fat^2^	34.8 + 1.1	31.0 + 1.1	31.7 + 1.1	32.0 + 1.1	*p* = 0.017
Dietary Quality (HEI‐2010 Total Score)^3^	49.9 + 1.7	56.6 + 1.8	54.1 + 1.7	54.8 + 1.8	*p* = 0.028

1 Least squares means controlling for weight, weight change, and race/ethnicity.

2 Change in % energy from fat from baseline to posttreatment in BWL: *p* = 0.005; in ME: *p* = 0.88.

3 Change in total HEI‐2010 scores from baseline to posttreatment in BWL: *p* = 0.009; *p* = 0.90.

### 3.3 Changes in diet, migraine characteristics, and weight

To understand how dietary changes related to changes in migraine characteristics, associations of reported changes in energy intake, percent energy from fat and diet quality with migraine characteristics (ie, number of migraine days, average migraine attack duration, and pain intensity), and weight loss were examined in BWL participants only. ME participants were not included in this analysis, as they did not receive dietary intervention targets and did not significantly change their diet. As shown in **Table** 3 **,** dietary changes were not significantly associated with changes in migraine characteristics (*p*'s > .05). Unexpectedly, reduced total energy intake and percent energy from fat were not associated with weight change.

**Table 3:** Effect of changes in dietary intake on changes in monthly migraine days (episodes), migraine duration, max pain severity, and weight in women with migraine and obesity who completed the behavioral weight loss (BWL) intervention as part of the Women's Health and Migraine trial (n = 41)

	Total energy intake (100 kcals/day)	% Energy from fat	HEI‐2010 total score (out of 100)
	*b‐value (p‐value)*		
*Monthly migraine Days* 1	0.22 (0.323)	−0.12 (0.314)	0.09 (0.281)
*Mean daily migraine duration (Hours)* 1	0.65 (0.237)	−0.36 (0.237)	0.26 (0.204)
*Migraine max pain intensity* 1	0.18 (0.111)	−0.09 (0.141)	−0.05 (0.247)
*Weight (kg)* ^*2*^	0.21 (0.511)	−0.30 (0.068)	0.07 (0.555)

1 Linear associations were estimated in participants in BWL controlling for race/ethnicity (non‐Hispanic White versus all others), weight, and weight change.

2 Linear associations were estimated in participants in BWL controlling for race/ethnicity (non‐Hispanic White versus all others).

## 4 DISCUSSION

This study is the first to examine whether following a low‐calorie low‐fat diet in the context of a randomized controlled behavioral weight control trial concurrently improves migraine characteristics and weight in women with comorbid migraine and overweight/obesity. As expected, a low‐calorie low‐fat diet in the context of a BWL intervention produced reductions in fat intake and improved diet quality relative to baseline and in comparison to ME. Changes in energy intake, percent energy from fat, and diet quality did not relate to improvements in any of the migraine parameters or weight change. Only the relationship between change in energy from fat and weight change showed a trend toward significance.

The observed findings differ from those of a crossover trial in which following a low‐fat diet (<20% of energy from fat) was associated with significant reductions in the number and severity of migraine attacks.^23^ However, the latter study differed in that it included both males and females with normal to obese weight status. Individuals with migraine who also have overweight/obesity may face additional challenges in adhering to a low‐fat diet. Participants in the crossover trial reduced their fat intake to 23% of total energy intake, whereas fat intake among women in BWL averaged 31.0% of total energy intake posttreatment. Thus, participants in the BWL intervention may not have reduced their fat intake to a clinically meaningful level. Future research should consider what unique strategies this population may need to adhere to a more restrictive diet (ie, in coping with cravings or compensatory eating postmigraine attack).

Findings suggest that, while the BWL significantly improved diet quality, this change was not associated with weight loss. This was unexpected. In the DIETFITS study, a weight control trial in which 609 adults with overweight and obesity were randomly assigned to a *healthy* low‐fat or a *healthy* low‐carbohydrate diet; researchers found that weight loss was significantly related to both diet quality and dietary adherence.^35^ Null findings may be due to the fact that while BWL participants improved diet quality by 6.7 points, their overall diet quality was still relatively low at a total of 56.6 out of 100 points, which is similar to the national average of 58.1 out of 100 for women ages 20+ in the United States.^36^ Thus, to contribute to weight loss, diet quality may have to improve to more substantially.

In this randomized controlled trial using gold standard dietary and migraine assessment methods, there was no significant association between a low‐fat low‐calorie diet and improvements in migraine characteristics. This study was a post hoc exploratory analysis, such that there are a few explanations for the findings. First, women in BWL may not have reduced their fat intake to a clinically meaningful level. Moreover, while the BWL participants improved diet quality 6.7 points, it remained quite low at 56.6 out of 100 points, suggesting that further removal of foods that are of lower nutritional quality and also suspected migraine triggers (ie, alcohol, processed meats, and snacks high in sodium and unhealthy fats and sugars, etc) may be needed for migraine characteristics to improve. Finally, findings from weight loss trials support that adhering to a diet is more beneficial for weight loss than the diet approach (ie, low‐fat, low‐carbohydrate, etc) that is followed.^37^ This may be true for individuals with migraine, such that the most effective diet in improving migraine characteristics may be the one to which a patient can adhere.

This study has several strengths. First, the data come from a randomized controlled trial in which women with neurologist‐confirmed migraine and overweight/obesity received a specific dietary prescription or migraine education. Second, diet was assessed using three nonconsecutive 24‐hour diet recalls, which, while subject to measurement error, is the current gold standard in assessing dietary intake. The study also had noteworthy limitations. First, given the sample, the dietary data are subject to social desirability bias, as underreporting bias is more likely to occur in women and those who have overweight/obesity. This is evidenced by the fact that women in both BWL and ME reduced their reported energy intake by similar amounts (336.8 and 232.7 calories, respectively), while only women in BWL experienced a significant weight reduction. To address this, underreporters were identified, and a sensitivity analysis was completed to ensure that their inclusion did not affect the pattern of results. Second, despite efforts to ensure that the BWL intervention did not address migraine and that ME did not address diet, participants in both groups were aware that the study was testing the effects of BWL on migraine. While it cannot be ruled out that participants in both groups changed their diets in ways not consistent with their specific intervention, the differences in weight outcomes and changes in percent energy from fat and diet quality suggest that overlap was minimal. Finally, the primary trial was powered to detect significant between‐group differences of at least 3 migraine headache days/month with 0.80 power at posttreatment with n = 140 and ≤18% attrition at posttreatment; however, because this was a post hoc exploratory completers analysis, an *a priori* power analysis was not completed.

## 5 CONCLUSIONS

This study examined adherence to a low‐calorie low‐fat diet as part of a BWL intervention for women with comorbid migraine and overweight/obesity and whether dietary changes were associated with improvements in migraine and weight loss relative to a control intervention. While a significant reduction in percent energy from fat and improvement in diet quality were observed among women in BWL, dietary changes were not associated with improvements in migraine or weight loss. It is possible that larger dietary changes are needed to experience migraine improvements. Future research is needed to clarify the mechanistic link between diet, weight, and migraine in general to refine therapies that can be used to treat overweight/obesity and migraine concurrently.

## CONFLICTS OF INTEREST

DSB received funding from the American Headache Society; RBL receives funding from Allergan.

## FUNDING

The WHAM trial was supported by a grant from the National Institute of Neurological Disorders and Stroke (R01 NS077925, PI: Bond). Dr. Evans is funded by a grant from the National Institute of Diabetes and Digestive and Kidney Diseases (K01 DK110142).
